# Targeting the self-amplifying loop between ATF6 and SNAI1 to inhibit epithelial‒mesenchymal transition in fibrotic lesions

**DOI:** 10.7150/thno.109442

**Published:** 2025-07-11

**Authors:** Tong Wu, Yan Liu, Jiyuan Ma, Lingshan Lu, Liang Wang, Mengmei He, Zhuang Hao, Xiaomin Liu, Luning Zhang, Chao Zhao, Mengzhang Tao, Chao Zheng, Jian Zhou

**Affiliations:** 1 Department of Ophthalmology, Xijing Hospital, Eye Institute of Chinese PLA, Fourth Military Medical University, 710032 Xi'an, China.; 2 State Key Laboratory of Cancer Biology, Department of Medical Genetics and Developmental Biology, Fourth Military Medical University, 710032 Xi'an, China.; 3 Institute of Orthopedic Surgery, Xijing Hospital, Fourth Military Medical University, 710032 Xi'an, China.

**Keywords:** cataract, epithelial-mesenchymal transition, activating transcription factor 6, snail family transcription factors, melatonin

## Abstract

**Background:** The epithelial‒mesenchymal transition (EMT) decisively contributes to human diseases such as organ fibrosis and tumors. However, the molecular mechanism triggering EMT remains unclear.

**Methods:** We elucidated a novel self-amplifying feedback loop involving activating transcription factor 6 (ATF6) and Snail family transcriptional repressor 1 (SNAI1) and assessed its significant role in unfolded protein response (UPR)-dependent EMT. Multiple *in vivo* and *in vitro* models were applied, including mouse models of trauma and laser-induced lens injury and human lens epithelial explants from patients with senile cataracts. RNA sequencing was performed to comprehensively analyze the molecular mechanisms underlying UPR-dependent EMT, and heterozygous *Atf6* knockout mice provided insights into the UPR-EMT crosstalk. The direct interaction between ATF6 and SNAI1 was verified via dual-luciferase assays. ATF6 expression was inhibited using AAV-shATF6 and melatonin (MLT) treatment in rodent models and cell cultures, respectively. Slit-lamp imaging, immunostaining, and western blotting were performed to assess EMT inhibition.

**Results:** ATF6 expression was markedly upregulated in fibrotic cataracts, and *ATF6* overexpression was sufficient to induce EMT-like changes both *in vivo* and *in vitro*. Similarly, compared with wild-type control mice, heterozygous *Atf6* knockout mice presented ameliorated injury-induced EMT. Dual-luciferase assays combined with functional studies revealed a self-amplifying loop between ATF6 and *SNAI1* that drives the uncontrollable progression of UPR-dependent EMT. Notably, MLT emerged as an effective inhibitor of UPR-dependent EMT and mitigated EMT-like alterations in parallel with *Atf6* knockdown, suggesting that MLT could be leveraged to target the ATF6-SNAI1 self-amplifying loop and inhibit EMT in human diseases.

**Conclusions:** Collectively, the results of this work demonstrate that the ATF6-SNAI1 self-amplifying loop acts as an important mediator of EMT and that MLT could be leveraged to target this loop and inhibit EMT in lens fibrosis.

## Introduction

The epithelial-mesenchymal transition (EMT) is a biological process that endows cells with mesenchymal features. EMT is crucial for embryogenesis, wound healing, and fibrosis 1. Revealing the cellular origin of myofibroblasts in most fibrotic tissues is challenging; thus, the murine lens has been proposed as an optional tool for investigating the EMT process 2, 3. Because the lens capsule isolates lens cells from other cell types, the lens epithelial cells (LECs) lining the inner surface of the lens capsule are readily identified, representing the major cell type undergoing EMT and leading to fibrotic cataracts 4. Given the advantages mentioned above, the murine lens is a powerful model organ for investigating the pathophysiology and molecular biology underlying EMT. Clinically, fibrotic cataracts, such as anterior subcapsular cataract (ASC) and posterior capsule opacification (PCO), represent the pathological EMT of lens epithelial cells 5. PCO is the most common cause of secondary vision loss in patients after cataract surgery 6. In the present study, we employed a murine lens injury model and human lenses to mimic pathological EMT phenotypes, investigate the molecular underpinnings involved, and assess potential treatment strategies for fibrosis.

The hallmark of EMT is the activation of EMT transcription factors (TFs) under different pathophysiological contexts. TFs suppress the expression of epithelial markers, such as E-cadherin and ZO-1, and activate the expression of mesenchymal markers, such as α-smooth muscle actin (α-SMA) and fibronectin 7. During EMT, SNAI1 is one of the most important TFs, which blocks E-cadherin expression by binding to its specific 5′-CACCTG-3′ element in the promoter 8. As a master TF of EMT, SNAI1 has emerged as an attractive target for the development of novel pharmaceutical approaches for treating EMT-related diseases 9. Although some inhibitory molecules have been reported to efficiently target SNAI1/p53 or E-cadherin interactions, SNAI1 is still considered an undruggable target 10, 11. Targeting the upstream molecular triggers of SNAI1 to initiate the EMT process could be a more feasible alternative.

The unfolded protein response (UPR) is a potential upstream signal of EMT initiation. Mechanical injury, such as cataract surgery and other physiological or pathological perturbations, may result in endoplasmic reticulum (ER) stress 12. The UPR initiates adaptive machinery to relieve ER stress and restore ER proteostasis. In general, stressors trigger the UPR via three stress sensors in the ER membrane: protein kinase R-like ER kinase (PERK), inositol requiring enzyme 1α (IRE1), and activating transcription factor 6 (ATF6) 13. Recent studies have further demonstrated the contribution of the UPR and its constituent components to changes in cell fate, causing aberrant cell fate reprogramming 14, 15. EMT, one of the most important and common types of cell fate conversion, is likely involved; activating the UPR directly induces spindle-like morphological changes in LECs, implicating an interplay between the UPR and EMT 16. However, the exact molecular mechanism underlying this interaction remains unknown.

Here, by employing a murine lens injury model and genetically modified mice and performing functional assays, we revealed a positive feedback loop between the ATF6 arm of the UPR and the EMT master TF SNAI1, which is sufficient to trigger and promote the development of UPR-dependent EMT. Moreover, by targeting UPR-dependent EMT, melatonin, a natural hormone, emerged as a promising therapeutic approach to EMT inhibition. These findings provide translational insights for developing novel therapeutic approaches to treat EMT-related disorders, including organ fibrosis and tumors.

## Results

### The ATF6 arm of the UPR pathway is activated in a lens EMT model

**Figure 1:**
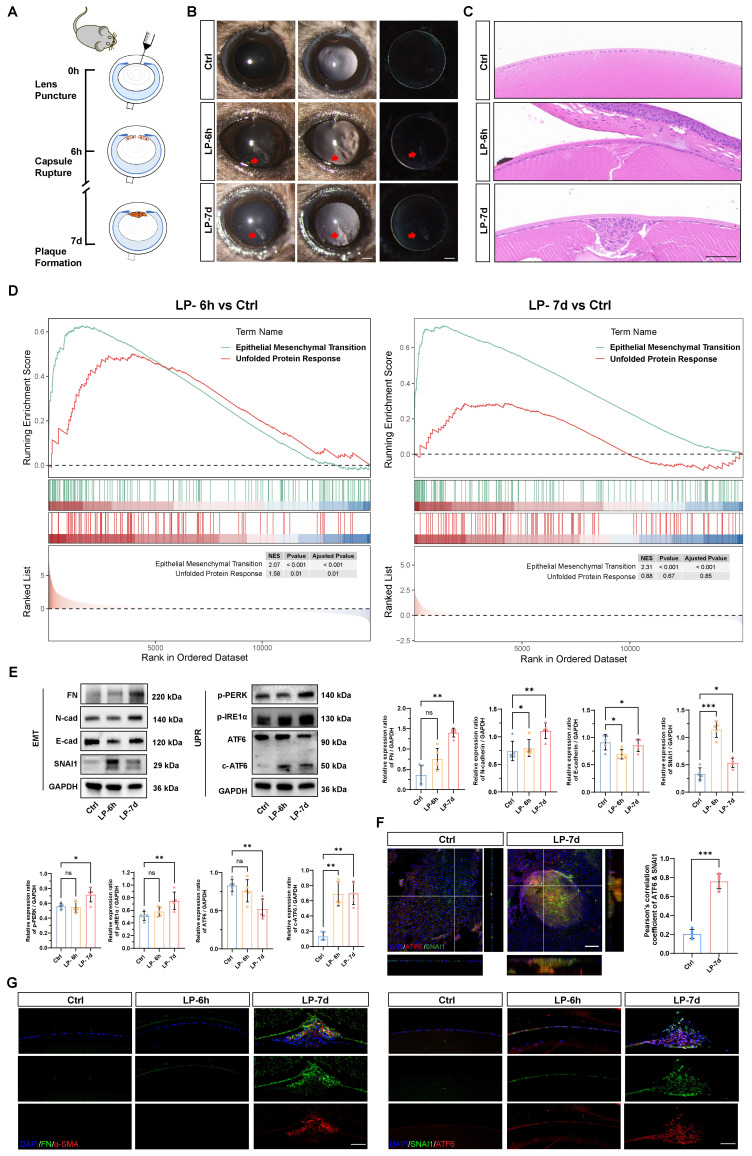
** The ATF6 arm of the UPR pathway is activated in a lens EMT model.** (A) Experimental design. (B) Images under a slit-lamp microscope of adult control mice, lens-punctured mice at 6 h, and lens-punctured mice at 7 d. Scale bars, 200 μm. (C) Representative images of HE-stained lens sections from adult control mice 6 h after lens puncture and 7 d after lens puncture. Scale bars, 50 μm. (D) Results of GSEA enrichment. (E) Western blotting and quantification of the EMT markers fibronectin (FN), N-cadherin (N-cad), and SNAI1; the epithelial cell marker E-cadherin (E-cad); and the UPR markers p-PERK, p-IRE1α, full-length ATF6 (ATF6), cleaved ATF6 (c-ATF6) (n = 5). Data are presented as mean ± SEM; ^*^p < 0.05, ^**^p < 0.01, ^***^p < 0.001. (F) Whole-mount immunostaining and 3D cross-sectional images of ATF6 and SNAI1. Quantitative colocalization analysis of immunostaining for ATF6 and SNAI1 by Pearson's correlation coefficient (n = 5). Scale bars, 100 μm. Data are presented as mean ± SEM; ^*^p < 0.05, ^**^p < 0.01, ^***^p < 0.001. (G) Representative images of immunostained lens sections of EMT markers, including FN, α-SMA, SNAI1, and ATF6, from adult control mice 6 h after lens puncture and 7 d after lens puncture. Scale bars, 50 μm.

To model the development of EMT* in vivo*, we generated a puncture injury on the mouse anterior lens capsule (Figure 1A). Six hours post injury, subcapsular epithelial cells at the injury site were absent, accompanied by disruption of continuous lens fibers. At 7 d post injury, LECs migrated and transformed into multicellular masses and subcapsular plaques (Figure 1B-C) 17. As expected, lens opacity due to a focal subcapsular cellular mass was induced in the lens puncture (LP) 7 d group, whereas the lenses remained transparent in the control group (Figure 1B). Hematoxylin and eosin (HE) staining of lens sections demonstrated distinct epithelial-mesenchymal transition (EMT) features, including the formation of subcapsular plaques composed of multilayered fibroblast-like cells, observed at 7 days post-injury (Figure 1C). Bulk mRNA sequencing was performed with control and punctured lenses at 6 h and 7 d post-injury to investigate the molecular mechanism driving epithelial cell EMT. The gene set enrichment analysis (GSEA) revealed that the EMT signaling pathway was expectedly upregulated in the lenses of the punctured mice at both 6 h and 7 d compared with the controls, confirming that EMT was successfully induced in the lens puncture model (Figure 1D). Moreover, we detected a concomitant increase in gene expression enriched in the UPR, indicating its' potential contribution to EMT pathology (Figure 1D and Figure S1A). Electron microscopy revealed ER extension in the LECs of the LP group, revealing the pathological hallmarks of ER stress (Figure S1B). Consistent with the findings of previous studies, the expression of EMT key transcription factor SNAI1 began to increase at 6 hours, and EMT markers, such as fibronectin (FN) and N-cadherin, was significantly upregulated in 7 d at the protein level, whereas, the epithelial cell marker E-cadherin was downregulated in 6 h and 7 d groups (Figure 1E) 17. To assess UPR activation in the lens EMT model, we assessed the expression of putative UPR readouts, including full-length ATF6 (ATF6), cleaved ATF6 (c-ATF6), p-PERK, and p-IRE1α, in the injured lenses. Notably, although the expression of all UPR signaling proteins was upregulated in 7 d post-injury lenses, the ATF6 arm of the UPR pathway was the only significantly activated machinery among the three UPR arms in 6 h post-injury lenses, as evidenced by the significant increase of cleaved ATF6 protein (Figure 1E). Moreover, we evaluated the expression of the master transcription factors ATF6 and SNAI1 in the UPR and EMT. Notably, whole-mount staining analysis of the lens anterior capsule demonstrated significant colocalization of ATF6 and SNAI1 within subcapsular plaques of injured lenses. In contrast, both proteins exhibited negligible expression in the single-layered epithelium of control lenses. (Figure 1F and Figure S1C). Immunostaining of FN and α‑SMA revealed high expression in 7 d post injury subcapsular plaques indicating the development of EMT. Immunostaining of lens cryosections revealed that six hours after lens injury, ATF6 and SNAI1 was detected in lens epithelial cells around the injury site. At 7 days, ATF6 and SNAI1 were highly expressed in the subcapsular plaque region enriched with FNs (Figure 1G). Taken together, these findings clearly demonstrated that the UPR and EMT are concomitantly activated, suggesting that an interaction between the UPR and EMT may be involved in EMT onset and progression.

### Activation of the UPR drives EMT in human lens epithelial cells

**Figure 2:**
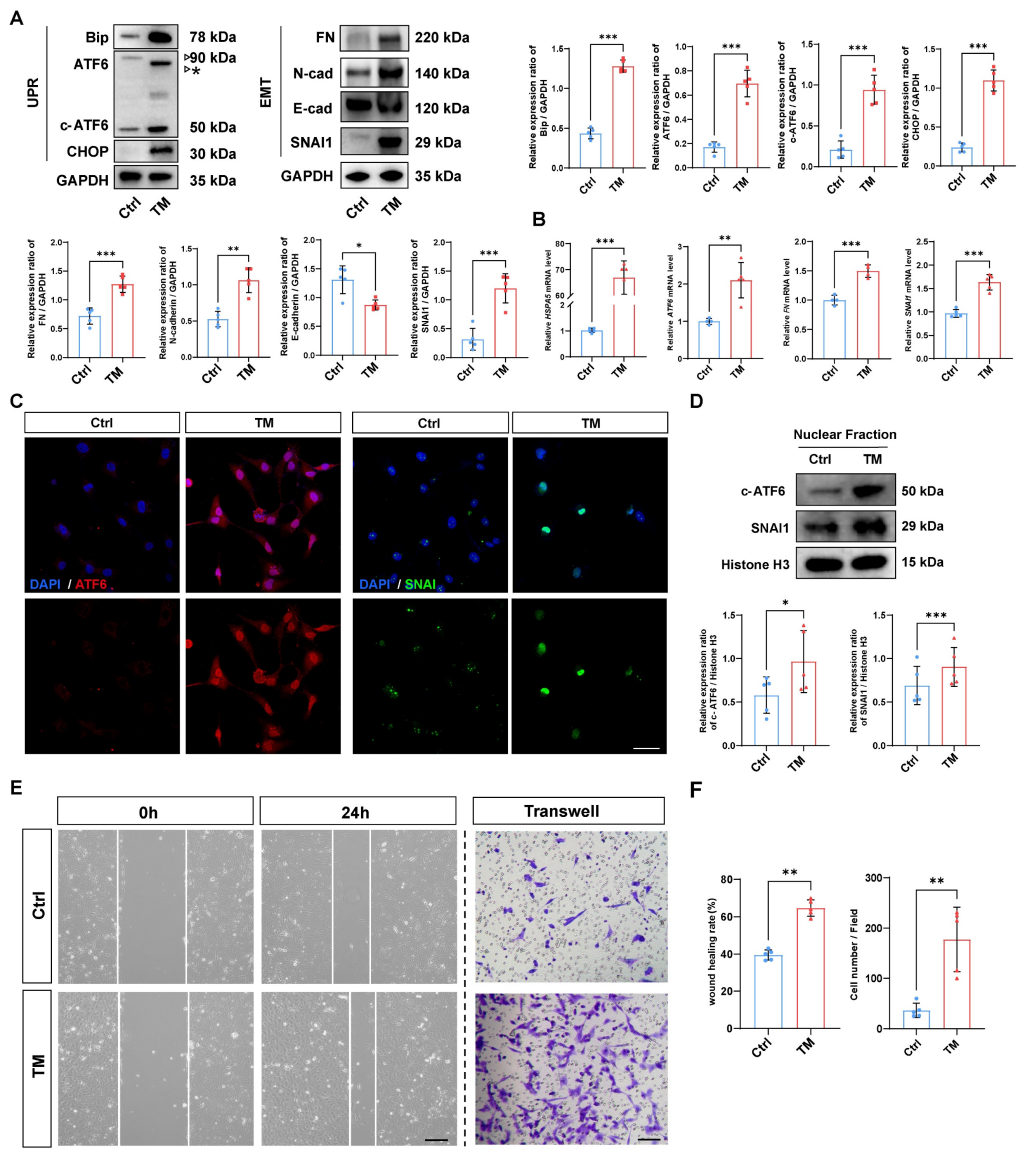
** Activation of the UPR drives EMT in human lens epithelial cells.** (A) Western blotting and quantification of the UPR markers BIP, CHOP, ATF6, and c-ATF6; the EMT markers FN, N-cadherin, and SNAI1; and the epithelial cell marker E-cadherin (n = 5). The asterisk denotes an unglycosylated form of full-length ATF6 due to TM treatment. Data are presented as mean ± SEM; ^*^p < 0.05, ^**^p < 0.01, ^***^p < 0.001. (B) qPCR and quantification of *HSPA5, ATF6, FN,* and *SNAI1* expression (n = 5). Data are presented as mean ± SEM; ^*^p < 0.05, ^**^p < 0.01, ^***^p < 0.001. (C) Immunofluorescence staining of ATF6 and SNAI1. Scale bars, 50 μm. (D) Nuclear protein fraction analysis (n = 5). Data are presented as mean ± SEM; ^*^p < 0.05, ^**^p < 0.01, ^***^p < 0.001. (E) Migration of HLE-B3 cells stimulated with TM was assessed using wound healing and transwell assays. Scale bars, 50 μm. (F) Quantification of wound closure and the number of migrating cells (n = 5). Data are presented as mean ± SEM; ^*^p < 0.05, ^**^p < 0.01, ^***^p < 0.001.

Given that activation of the UPR has been reported to induce the transformation of cell fate, we investigated whether activation of the UPR alone is sufficient to initiate EMT 18. To that end, a UPR inducer, tunicamycin (TM), was used to rapidly induce the UPR 19. As expected, western blotting demonstrated that TM effectively upregulated the expression of the UPR markers BIP and CHOP in HLE-B3 cells, and qPCR analyses also confirmed that TM upregulated the mRNA expression of UPR markers *HSPA5* and *ATF6*. Meanwhile, TM treatment induced the accumulation of unglycosylated full-length ATF6, along with an increase in cleaved ATF6 protein (Figure 2A-B). Surprisingly, along with the UPR activation induced by TM, the EMT markers fibronectin (*FN*), N-cadherin, and SNAI1 were also significantly upregulated, and the expression of the epithelial cell marker E-cadherin significantly decreased upon UPR activation (Figure 2A-B). Given that both ATF6 and SNAI1 are master TFs that translocate into the nucleus and transactivate UPR- and EMT-related genes, we used immunofluorescence staining and nuclear protein fraction analysis to further verify the nuclear translocation of ATF6 and SNAI1 upon UPR activation. The results revealed simultaneous increases in the nuclear translocation of ATF6 and SNAI1 following TM addition (Figure 2C-D). Moreover, after TM treatment, cell migration markedly increased in the HLE-B3 cells, but not in the controls, concordantly favoring EMT-related cellular behavior (Figure 2E-F). Collectively, our data suggest that the activation of the UPR is sufficient to induce EMT in human LECs.

### Modulating ATF6 expression is sufficient to affect EMT outcomes* in vivo*

**Figure 3:**
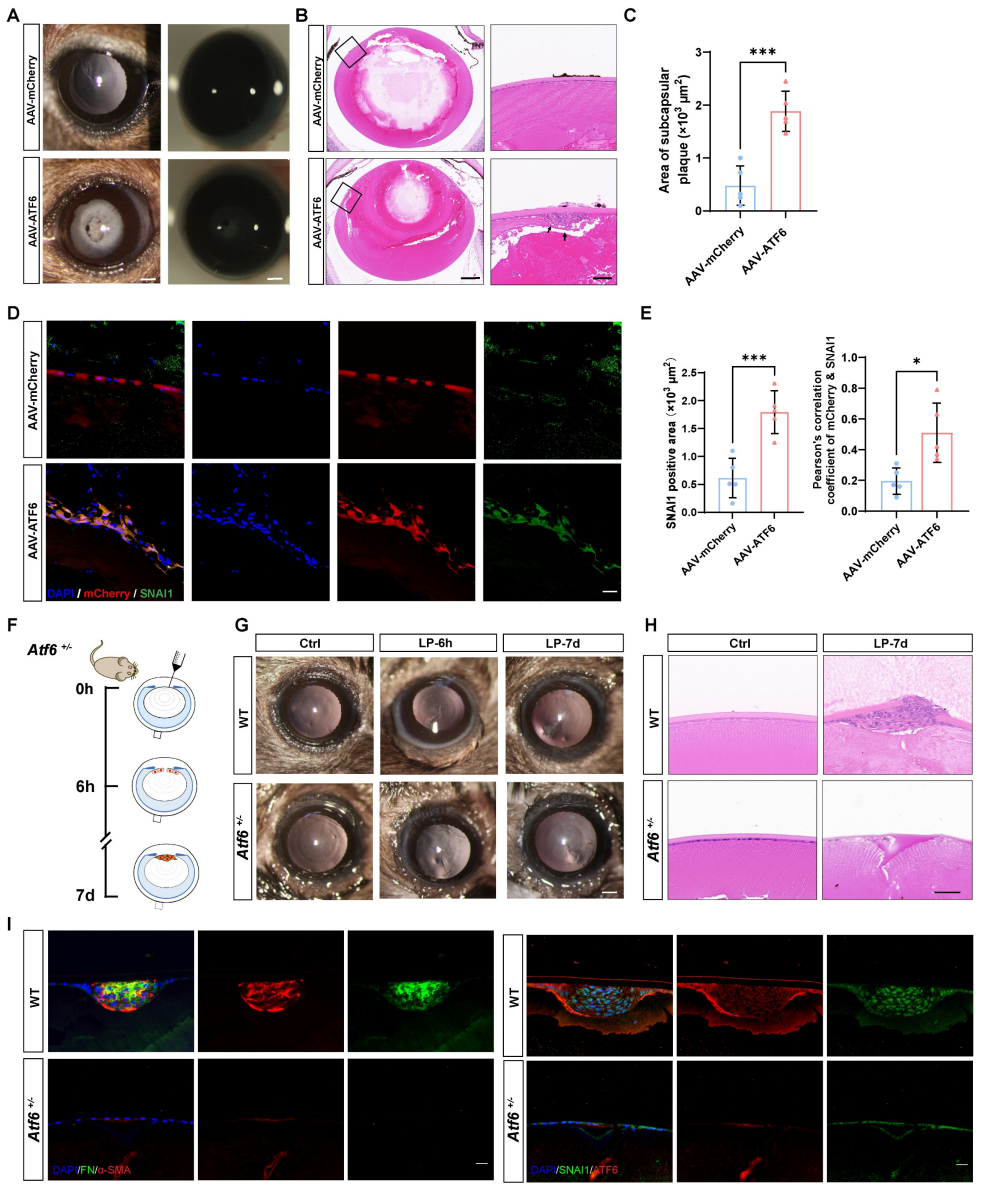
** ATF6 regulates lens epithelial cell EMT* in vivo*.** (A) Images of AAV-mCherry-infected mouse lenses and AAV-ATF6-infected mouse lenses under a slit-lamp microscope. Scale bars, 200 μm. (B) Representative images of HE-stained sections of AAV-mCherry-infected mouse lenses and AAV-ATF6-infected mouse lenses. Scale bars, 200 μm and 50 μm. (C) Quantification of the area of subcapsular plaque (n = 5). Data are presented as mean ± SEM; ^*^p < 0.05, ^**^p < 0.01, ^***^p < 0.001. (D) Immunofluorescence staining of the EMT marker SNAI1 and colocalization of mCherry and SNAI1 in lens sections. Scale bars, 25 μm. (E) Quantification of SNAI1-positive area (n = 5). Quantitative colocalization analysis of immunostaining for ATF6 and SNAI1 by Pearson's correlation coefficient (n = 5). Data are presented as mean ± SEM; ^*^p < 0.05, ^**^p < 0.01, ^***^p < 0.001. (F) Experimental design. (G) Images of lenses at 7 d after injury in wild-type (WT) mice and *Atf6^+/-^* mice under a slit-lamp microscope. Scale bars, 200 μm. (H) Representative images of HE-stained sections 7 d after lens puncture from WT and *Atf6^+/-^* mice. Scale bars, 50 μm. (I) Immunofluorescence staining of the EMT markers α-SMA, FN, SNAI1, and ATF6 in murine lens sections. Scale bars, 50 μm.

Our evidence that the preferential activation of the ATF6 arm of the UPR in the injury-induced EMT model, together with the fact that the UPR is sufficient to drive EMT development, leads to the intriguing speculation that stressed epithelial cells may coopt the ATF6-dependent UPR machinery to initiate EMT. To test this hypothesis, we forced the expression of ATF6 in mouse lenses to mimic preferential activation of the ATF6 arm and investigated whether ATF6-expressing LECs recapitulate the EMT phenotype *in vivo*. It was observed from eye sections intracamerally injected with adeno-associated virus (AAV)-GFP of different serotypes that only type 2/DJ (AAV2/DJ) could efficiently infect LECs (Figure S2A). We generated expression constructs encoding a fragment of the ATF6 (39-373) sequence to ensure effective nuclear translocation of the exogenous ATF6 protein (Figure S2B), as previously reported 16. AAV2/DJ-CMV-mCherry-ATF6 (39-373) (AAV-ATF6) and AAV2/DJ-CMV-mCherry (AAV-mCherry) were used in the subsequent experiments. One month after infection, severe lens opacity observed via slit-lamp microscopy was present in the AAV-ATF6 group but not in the AAV-mCherry group (Figure 3A). In line with these findings, HE staining revealed a subcapsular multicellular mass in the AAV-ATF6-infected lenses instead of the single-layered epithelial cells observed in the control lenses (Figure 3B-C). Furthermore, immunofluorescence staining of lens sections revealed that SNAI1 was highly expressed in subcapsular plaques and colocalized with the fluorescent reporter of AAV-ATF6 (Figure 3D-E). These *in vivo* data indicate that ATF6 alone is sufficient to drive EMT and strongly suggest that interplay between two master transcription factors, ATF6 and SNAI1, initiates EMT.

To confirm that ATF6 plays a pivotal role in EMT, we performed lens puncture injury in *Atf6* heterozygous knockout (*Atf6^+/-^*) and wild-type (WT) mice to investigate whether such a minor dosage of genetic ablation would perturb the disease outcome (Figure 3F). Adult *Atf6^+/-^* mice (6-8 weeks old) presented no apparent ocular structural abnormalities (Figure S3A), as previously reported 20. Examination under a slit-lamp microscope at 6 h after injury revealed damage to and rupture of the anterior lens capsules in both *Atf6^+/-^* and WT mice. By day 7, the capsular lesion had healed, resulting in localized subcapsular opacity (Figure 3G and Figure S3B). HE staining of lens sections revealed typical signs of EMT in fibroblastic cell clusters subsequent to capsular injury (Figure 3G). Conversely, in *Atf6^+/-^* mice, the injured region exhibited homogeneous changes devoid of cellular proliferation or aggregation (Figure 3H). In sharp contrast to the injured region enriched for FN and α-SMA expression in wild-type lenses, little to no expression of EMT markers was observed in *Atf6^+/--^* mice. Concordantly, immunofluorescence staining of SNAI1 and ATF6 revealed few positive signals in* Atf6^+/-^* lenses and substantial signals in the injured region of wild-type lenses (Figure 3I). Taken together, these findings provide genetic evidence supporting a critical role for ATF6 in EMT development.

### A self-amplifying loop between ATF6 and SNAI1 promotes EMT

**Figure 4:**
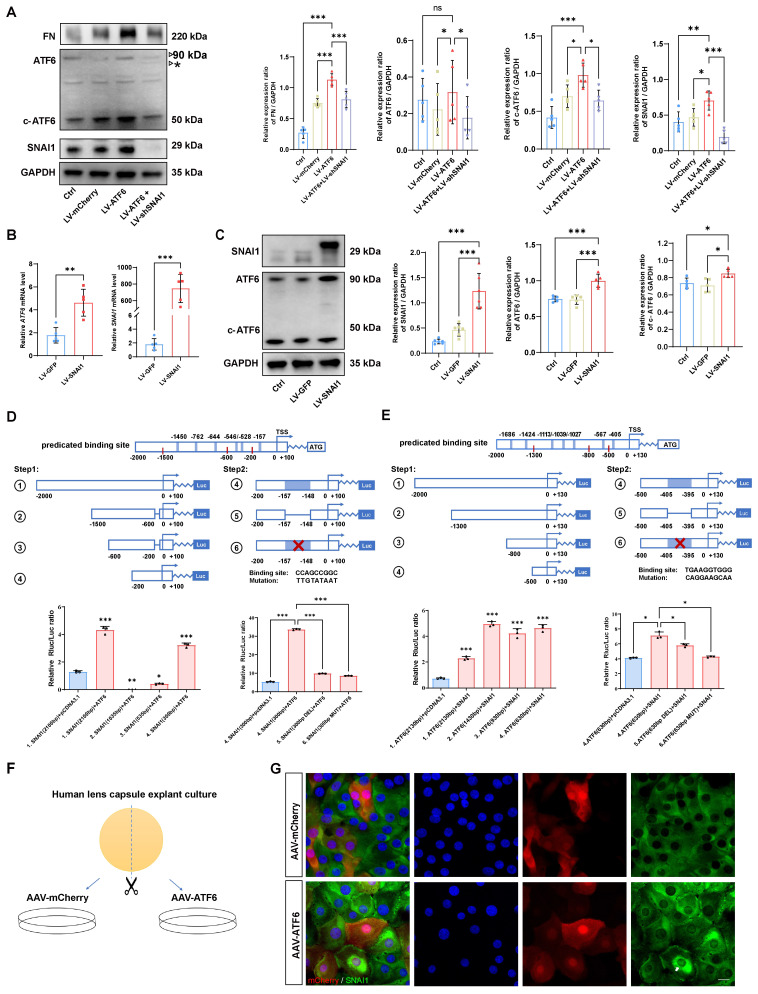
** The ATF6-SNAI1 self-amplifying loop drives EMT *in vitro*.** (A) FN, ATF6, c-ATF6, and SNAI1 expression in the control, LV-mCherry, LV-ATF6 and LV-ATF6+LV-shSNAI1 groups, were subjected to western blot analyses. Protein expression was quantified (n = 5). Data are presented as mean ± SEM; ^*^p < 0.05, ^**^ p < 0.01, ^***^ p < 0.001. (B, C) Quantification of the ATF6 and SNAI1 expression by qPCR and western blotting. Data are presented as mean ± SEM; ^*^p < 0.05, ^**^p < 0.01, ^***^p < 0.001. (D) Luciferase assay experimental design and expression in 293T cells. No. 1 refers to [-2000/+100] *SNAI1*, No. 2 refers to [-1500/-600] *SNAI1*, No. 3 refers to [-600/-200] *SNAI1*, No. 4 refers to [-200/+100] *SNAI1*, No. 5 refers to [-200/+100 Delate] *SNAI1* and No. 6 refers to [-200/+100 Mutant] *SNAI1* (n = 3)*.* Data are presented as mean ± SEM; ^*^p < 0.05, ^**^p < 0.01, ^***^p < 0.001. (E) Luciferase assay experimental design and expression in 293T cells. No. 1 indicates [-2000/+130] *ATF6*, No. 2 indicates [-1300/+130] *ATF6*, No. 3 indicates [-800/+130] *ATF6*, No. 4 indicates [-500/+130] *ATF6* (n = 3)*.* No. 5 refers to [-500/+100 Delate] *ATF6* and No. 6 refers to [-500/+100 Mutant] *ATF6* (n = 3)*.* Data are presented as mean ± SEM; ^*^p < 0.05, ^**^p < 0.01, ^***^p < 0.001 (F) Study design for human lens capsule explant culture. (G) Immunofluorescence staining of SNAI1 in AAV-mCherry-infected human lens capsules and AAV-ATF6-infected human lens capsules. Arrow indicates nuclear fluorescent signals of SNAI1. Scale bars, 25 μm.

Given the tightly intertwined expression of ATF6 and SNAI1 in stressed HLE-B3 cells and injured or AAV-ATF6-infected lenses, we further determined whether direct transcriptional regulation between ATF6 and SNAI1 would mediate UPR-dependent EMT. First, we forced the expression of ATF6 in HLE-B3 cells with Lenti-ATF6-overexpressing virus (LV-ATF6) and assessed the expression of ATF6, c-ATF6, SNAI1, and FN. As expected, LV-ATF6 infection successfully increased the level of c-ATF6 protein. Notably, SNAI1 and FN levels were also significantly enhanced upon ATF6 overexpression. When ATF6-overexpressing HLE-B3 cells were infected with the LV-shSNAI1 virus, we observed a decrease in not only SNAI1 expression but also ATF6 and c-ATF6 expression (Figure 4A). Second, we infected HLE-B3 cells with an LV-SNAI1-overexpressing virus and assessed the expression of ATF6 and SNAI1. Intriguingly, western blotting and qPCR analyses indicated that SNAI1 overexpression led to significant upregulation of ATF6 and c-ATF6 expression (Figure 4 B-C). Taken together, these results indicate that ATF6 and SNAI1 can promote each other's expression, accounting for their tightly intertwined expression in stressed HLE-B3 cells and injured or AAV-ATF6-infected lenses.

These data prompted us to investigate whether ATF6, a potent stress TF, could directly transactivate SNAI1. To this end, we queried the TRANSFAC database and identified 5 potential binding sites of ATF6 in the SNAI1 promoter region (Table S1). To include 5 predicted ATF6 binding sites, we split the total regulatory region before the start codon ATG of mouse SNAI1 gene into four sections, namely, 1.[-2000/+100] *SNAI1*, 2.[-1500/-600] *SNAI1*, 3.[-600/-200] *SNAI1*, and 4.[-200/+100] *SNAI1*), which were co-transfected with pCMV-ATF6 vector. A luciferase reporter assay revealed that nucleotide (nt)s from -1500 to -200 had little effect on ATF6-induced SNAI1 promoter activity. However, 4.[-200/+100] *SNAI1* significantly increased ATF6-induced *SNAI1* promoter activity versus that in the control, indicating that this region is critical for ATF6 activation of the *SNAI1* promoter. Interestingly, the -200/+100 construct contains only one predicted binding sequence (CCAGCCGGC, -157 from the transcription start site), suggesting direct transactivation of SNAI1 by the binding of ATF6 to this element. To further confirm the presence of the ATF6 response element, deletion and mutation constructs between -157 and -148 were constructed and cotransfected with pCMV-ATF6 into 293T cells. Compared with transfection with 4.[-200/+100], transfection with deleted or mutant constructs significantly reduced the luciferase activity (Figure 4D). These findings indicate that ATF6 can directly transactivate SNAI1 by binding to the element [-157/-148].

SNAI1 is a core EMT transcription factor, and whether SNAI1 can directly transactivate ATF6 is unclear. To include 7 predicted SNAI1 binding sites (Table S2), we designed a series of *ATF6* promoter constructs (1. [-2000/+130] *ATF6*, 2. [-1300/+130] *ATF6*, 3. [-800/+130] *ATF6*, *4.* [-500/+130] *ATF6)* and cotransfected them with *SNAI1* into the 293T cell line. When SNAI1 was overexpressed, the relative luciferase activity of all aforementioned ATF6 constructs showed significant elevation compared to the vector control (Figure 4E), indicating that all these constructs contain SNAI1-responsive elements. Notably, the [-500/+130] construct (the shortest variant) harbors a single predicted binding motif (TCAGGT) located at position -405 relative to the transcription start site (Table S2). To further confirm whether SNAI1-mediated transactivation of ATF6 occurs through this potential cis-regulatory element, deletion and mutation constructs between -405 and -395 were constructed and cotransfected with pCMV-SNAI1 into cells. Compared with transfection with 4.[-500/+130], transfection with deleted or mutant constructs significantly reduced the luciferase activity (Figure 4E). Collectively, these results strongly suggest a self-amplifying loop between ATF6 and SNAI1, which might mediate UPR-dependent EMT. To confirm these findings in human tissue, human anterior lens capsules were divided into two groups and cultured with AAV-ATF6 or AAV-mCherry infection (Figure 4F). Consistently, the immunofluorescence staining results indicated that the nuclear translocation of *SNAI1* was significantly greater in the AAV-ATF6 group than in the AAV-mCherry group (Figure 4G).

### MLT inhibits EMT by stunting the self-amplifying loop between ATF6 and SNAI1

**Figure 5:**
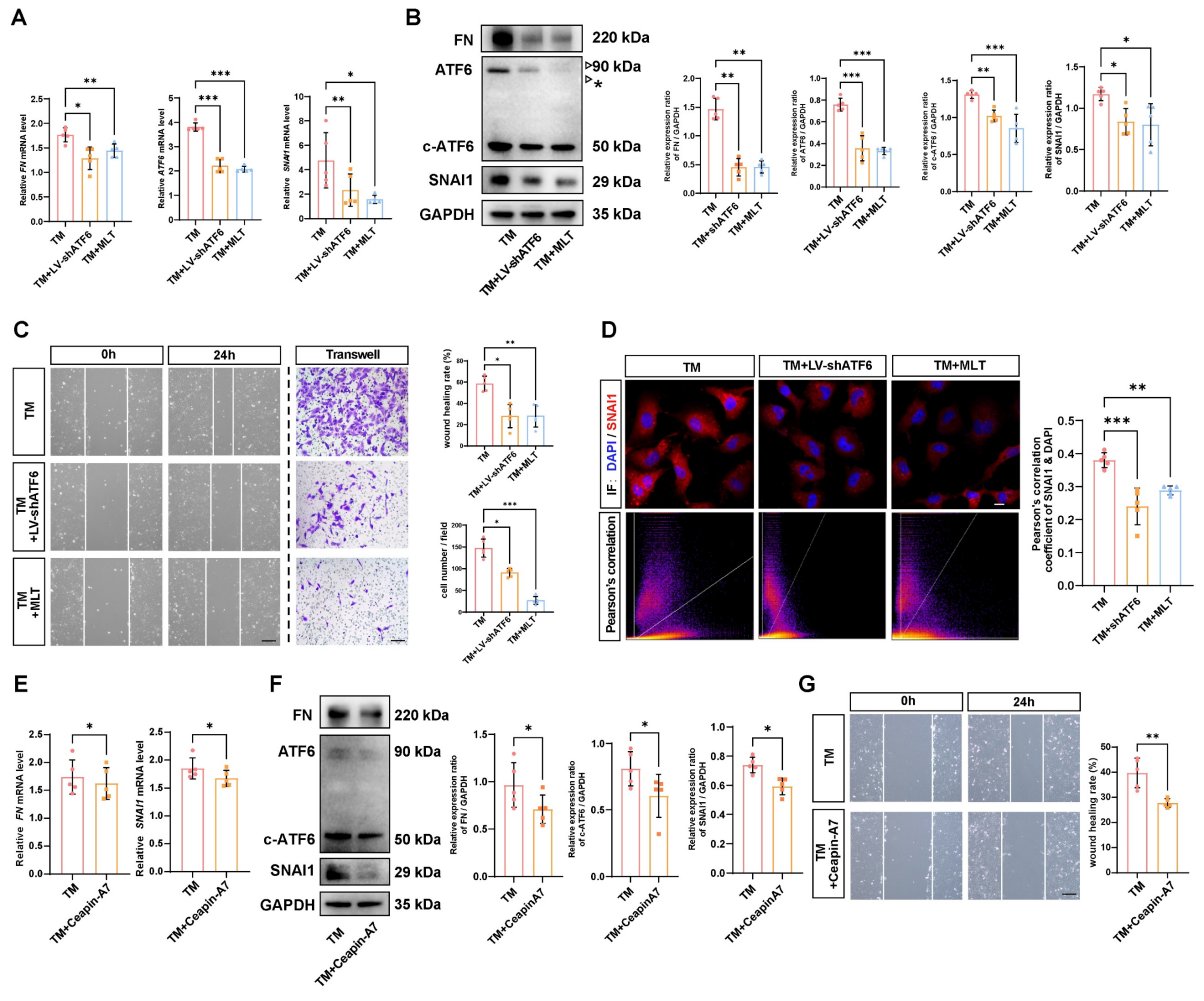
** Effects of inhibiting ATF6 activation on EMT-like cell behaviors *in vitro*.** (A) qPCR and quantification of ATF6, FN and SNAI1 expression (n = 5). Data are presented as mean ± SEM; ^*^p < 0.05, ^**^p < 0.01, ^***^p < 0.001. (B) Western blotting and analysis of the EMT markers FN, SNAI1, ATF6 and c-ATF6 (n = 5). The asterisk denotes an unglycosylated form of full-length ATF6 due to TM treatment. Data are presented as mean ± SEM; ^*^p < 0.05, ^**^p < 0.01, ^***^p < 0.001. (C) Migration ability of HLE-B3 cells in the TM, TM+LV-shATF6, and TM+MLT groups, as detected by wound healing and transwell assays. (n = 5). Scale bars, 50 μm. Quantification of wound closure and the number of migrating cells (n = 5). Data are presented as mean ± SEM; ^*^p < 0.05, ^**^p < 0.01, ^***^p < 0.001. (D) Immunofluorescence staining of SNAI1 in HLE-B3 cells in the TM, TM+LV-shATF6, and TM+MLT groups. Scale bars, 50 μm. Quantitative colocalization analysis of immunostaining for DAPI and SNAI1 by Pearson's correlation coefficients (n = 5). Data are presented as mean ± SEM; ^*^p < 0.05, ^**^p < 0.01, ^***^p < 0.001. (E) qPCR and quantification of FN and SNAI1 expression (n = 5). Data are presented as mean ± SEM; ^*^p < 0.05, ^**^p < 0.01, ^***^p < 0.001. (F) Western blotting and analysis of the EMT markers FN, SNAI1 and c-ATF6 (n = 5). Data are presented as mean ± SEM; ^*^p < 0.05, ^**^p < 0.01, ^***^p < 0.001. (G) Migration ability of HLE-B3 cells in the TM, TM+Ceapin-A7 groups, as detected by wound healing. (n = 5). Scale bars, 50 μm. Quantification of wound closure (n = 5). Data are presented as mean ± SEM; ^*^p < 0.05, ^**^p < 0.01, ^***^p < 0.001.

Delineating the molecular mechanisms underlying EMT has direct implications for therapeutic development, considering EMT inhibition could mitigate tissue fibrosis 21. Thus, we investigated whether the self-amplifying loop between ATF6 and SNAI1 could be leveraged to inhibit UPR-dependent EMT. It has been reported that MLT is a potent direct UPR pathway inhibitor, especially affecting the ATF6 branch 22. The time-course western blot analysis demonstrated that starting at 6 hours after TM treatment, the protein level of the ATF6 cleaved form in control group cells began to significantly upregulate, while in the melatonin-treated group, the protein levels of ATF6 cleaved form showed significant suppression compared to the control group at 12 and 24 hours (Figure S4A). Similarly, qPCR and western blot analyses indicated that MLT significantly downregulated ATF6 expression at the mRNA level and inhibited both ATF6 and c-ATF6 protein in stressed HLE-B3 cells. Both knockdown of ATF6 with LV-shATF6 and MLT treatment significantly decreased the expression of FN and SNAI1 (Figure 5A-B). Furthermore, cell migration assays revealed that both shATF6 and MLT treatment significantly ameliorated cell migration; in particular, MLT treatment decreased the number of migrated cells by more than 50% (Figure 5C). Immunofluorescence staining of the treated HLE-B3 cells revealed that both shATF6 and MLT treatment efficiently decreased the nuclear translocation of *SNAI1* (Figure 5D). To further substantiate that inhibiting ATF6 pathway activation effectively suppresses EMT, we included a specific ATF6 inhibitor Ceapin-A7 23. qPCR and Western blot results demonstrated that Ceapin-A7 significantly inhibited the production of cleaved ATF6, thereby further reducing the mRNA and protein expression levels of FN and SNAI1 (Figure 5E-F). Additionally, Ceapin-A7 suppressed the migratory capacity of HLE-B3 cells following TM treatment (Figure 5G). Together, these data strongly suggest that targeting activation of the ATF6 arm of UPR could be a therapeutic strategy to inhibit EMT.

**Figure 6:**
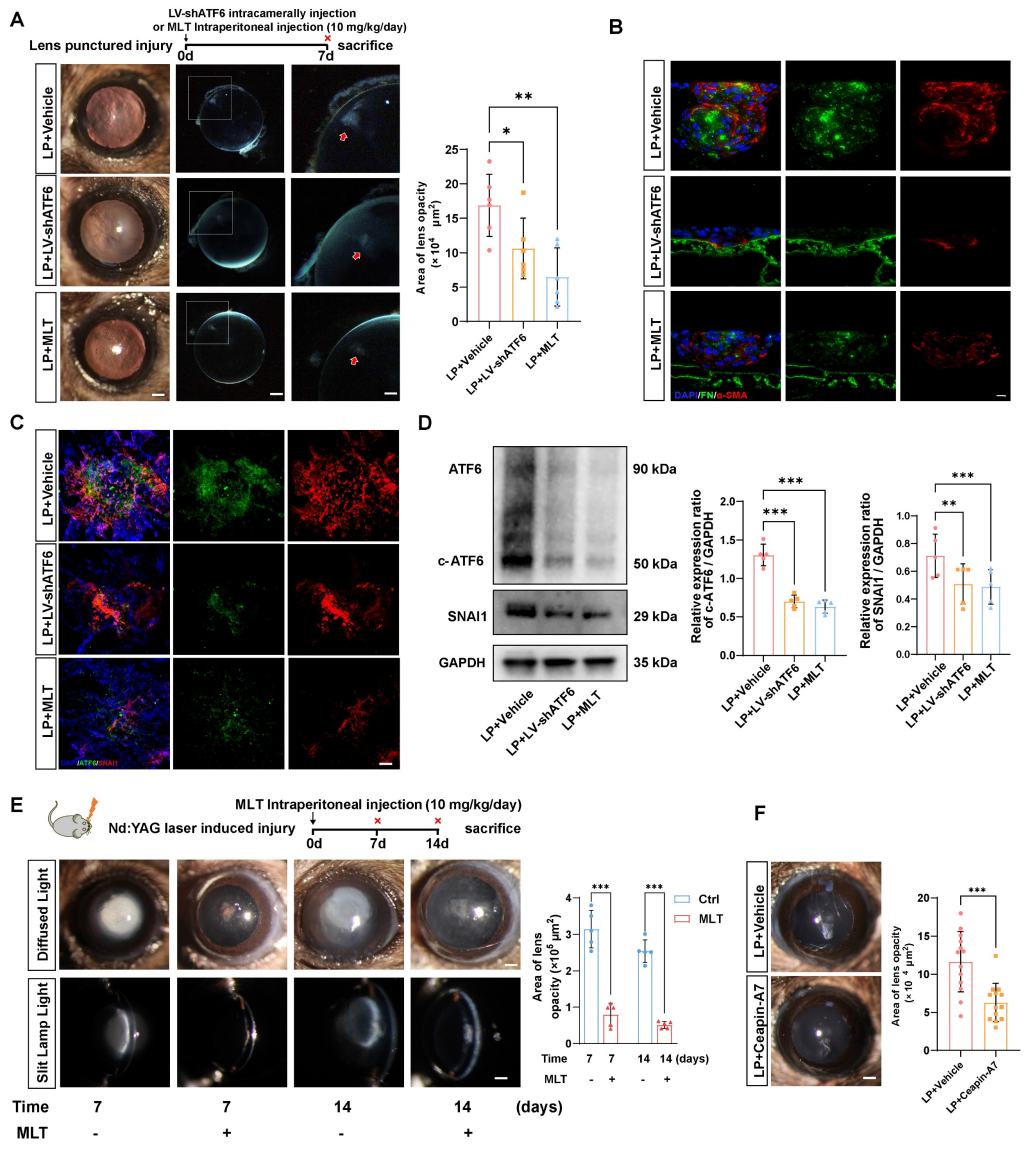
** Melatonin effectively mitigated EMT-like alterations in parallel with *Atf6* knockdown *in vivo*.** (A) Images of the lenses of LP mice, LP+LV-shATF6-treated mice, and LP+MLT mice under a slit lamp microscope. Scale bars, 200 & 500 μm. Quantification of the subcapsular plaque area (n = 6). Data are presented as mean ± SEM; ^*^p < 0.05, ^**^p < 0.01, ^***^p < 0.001. (B) Representative images of lens sections with immunofluorescence staining of α‑SMA and FN. Scale bars, 10 μm. (C) Whole-mount immunofluorescence staining for ATF6 and SNAI1 in the above groups. Scale bars, 50 μm. (D) Western blotting and quantification of the EMT markers SNAI1 and c-ATF6 (n = 5). Data are presented as mean ± SEM; ^*^p < 0.05, ^**^p < 0.01, ^***^p < 0.001. (E) Images of laser-induced lens-punctured mice treated with MLT for 7 or 14 d under a slit‒lamp microscope. Scale bars, 200 μm. Quantification of opacity area (n = 5). Data are presented as mean ± SEM; ^*^p < 0.05, ^**^p < 0.01, ^***^p < 0.001. (F) Images of the lenses of LP mice and LP+Ceapin-A7-treated mice under a slit lamp microscope. Scale bars, 200 μm. Quantification of opacity area (n = 13). Data are presented as mean ± SEM; ^*^p < 0.05, ^**^p < 0.01, ^***^p < 0.001.

Next, we verified whether the therapeutic effects of LV-shATF6, MLT and Ceapin-A7 hold true in an* in vivo* EMT model. We adopted a mouse model with puncture lens injury, and MLT was administered to the mice at a dose of 10 mg·kg^-1^·day^-1^, as previously reported 24, 25. Surprisingly, the area of lens subcapsular opacity in the LV-shATF6 and MLT groups was significantly lower than that in the vehicle-treated group (Figure 6A). Immunofluorescence staining of lens cryosections revealed that LV-shATF6 and MLT treatment apparently reduced the expression of α-SMA and FN (Figure 6B). Moreover, both immunofluorescence staining and western blotting results of the lens capsule revealed a decrease in ATF6 and SNAI1 expression after LV-shATF6 and MLT treatment (Figure 6 C-D). To rule out influences of infection or lens damage introduced by needle puncture, we adopted another mouse model with YAG laser-induced lens capsular injury. Compared with vehicle treatment, MLT treatment significantly ameliorated cataract formation at 7 and 14 d post injury (Figure 6E). In keeping with this, Ceapin-A7 treatment at a dose of 10 mg·kg^-1^·day^-1^ significantly decreased the area of lens opacification and apparently reduced the subcapsular multicellular mass in mice with puncture lens injury (Figure 6F and Figure S4B). Collectively, our data indicate that the clinically translatable ATF6 inhibitor is a promising therapeutic approach for EMT inhibition that acts by stunting the ATF6-SNAI1 self-amplifying loop.

## Discussion

The UPR is an adaptive mechanism that evolves to restore ER proteostasis upon ER stress. Accumulating evidence has revealed the crucial role of the IRE1 and PERK arms of the UPR in various pathological conditions 26, 27. However, research on the ATF6 branch is still in its infancy. Consistent with previous studies, our results further reinforced the important role of the UPR in pathological EMT in fibrotic cataracts in the early stage 28, 29. We found that the activation of the UPR stimulated by TM significantly improved the migration ability of lens epithelial cells, which is often associated with disease progression 30. Furthermore, of all three classical UPR branches, the activation of ATF6 was the most significantly affected 31, and the overexpression of ATF6 alone was sufficient to drive subcapsular plaque formation in the lens. These data strongly suggest that the UPR, especially the ATF6 branch, is an upstream signal of EMT initiation and progression. In addition to ATF6 branch, we also found activation of both IRE1α and PERK arms, which might be explained by extensive crosstalk among the three UPR branches 32. Notably, it has been reported ATF6-mediated transcriptional activation of XBP1, which subsequently enhances IRE1α-dependent UPR signaling 33. Interestingly, our dual-luciferase assays combined with functional studies revealed that ATF6 and SNAI1 could directly transactivate each other. Therefore, ATF6-mediated UPR activation is not only an upstream trigger for EMT but also a vicious positive feedback loop that drives uncontrollable EMT progression. Our findings provide new insight into the relationship between the UPR and EMT regulation. The development of small molecules and gene therapies targeting specific UPR components may offer potential therapeutic strategies for treating EMT-associated diseases.

A previous study revealed that the phenotype of *Atf6^-/-^* mice affects the retinal photoreceptor system at older ages without notable lens changes,^20^ indicating that ATF6 is dispensable for lens development and homeostasis. However, our results revealed that a minor dose of ATF6 ablation perturbed the EMT outcome, as evidenced by reduced formation of fibroblastic cell clusters and little to no upregulation of EMT markers. To our knowledge, this is the first study reporting that heterozygous knockout of *Atf6* attenuates EMT-like lesions *in vivo*. In line with these findings, previous studies using genetically modified mice identified ATF6 as a critical regulator in renal and pulmonary fibrosis pathogenesis, where genes transcriptionally controlled by the ATF6-dependent ER stress pathway elicited profibrogenic effects 34, 35. However, the present study has limitations, particularly the absence of lens-specific conditional knockout mouse models. Future study should include conditional ATF6 knockout mice to further validate ATF6's role in EMT, thereby allowing more precise dissection of ATF6's pathological mechanisms. Overall, our study and others support the inhibition of ATF6 transcriptional activity as a potential therapeutic strategy for mitigating EMT-induced fibrosis in various organs.

MLT, a natural hormone, has been shown to regulate multiple biological functions and exert therapeutic effects on various diseases, such as coronary heart disease, schizophrenia, chronic pain, and Alzheimer's disease 36-38. The antioxidative, apoptosis-inducing, anti-inflammatory and antiangiogenic properties of MLT improve disease outcomes 39, 40. Our study provides evidence supporting the anti-EMT effects of MLT in a murine lens injury model. In our lens injury mouse model mimicking the onset and progression of EMT, MLT treatment effectively downregulated the expression of ATF6 and inhibited its downstream target, SNAI1, significantly decreasing the volume of fibrotic plaques under the lens capsule. Furthermore, MLT inhibited the EMT-like behaviors of stressed HLE-B3 cells *in vitro*. Considering that MLT is a promising translatable drug, these data provide a valuable molecular basis for the future application of MLT in treating EMT-related diseases. While our study and others demonstrate MLT suppresses ATF6 expression/cleavage 41-44, its precise mechanisms require elucidation. Building on MLT's documented effects, two mechanistic hypotheses merit consideration and further study. MLT could modulate ATF6 via Phospholipase C/calcium signaling through receptor-mediated calcium homeostasis restoration 45, 46. Furthermore, calreticulin interacts with both MLT and ATF6 47, 48, and diminished ATF6-calreticulin binding enhances Golgi trafficking and cleavage of ATF6 48, proposing MLT might stabilize this binding to inhibit ATF6 cleavage.

In conclusion, our study revealed a novel self-amplifying loop between ATF6 and SNAI1 that initiates and promotes EMT in lens epithelia. These findings could be leveraged to develop therapeutic approaches to inhibit EMT in many human diseases, such as organ fibrosis and tumors. Moreover, our study also serves as a proof of concept that the murine lens injury model may have broader implications for understanding EMT-related organ fibrosis and testing therapeutic approaches for preventing this disease.

## Materials and Methods

### Mouse model of traumatic cataracts

The animal studies conformed to the guidelines for the care and use of laboratory animals per the ARVO Statement for the Use of Animals in Ophthalmic and Vision Research. All animal experiments were approved by the Animal Care and Use Committee of the Fourth Military Medical University (No. 20240474). Male *Atf6^+/-^* mice were purchased from Cyagen Biosciences (Guangdong, China). Male wild-type C57BL/6J mice were purchased from the Laboratory Animal Center, Fourth Military Medical University. The mice were housed in a specific pathogen-free, temperature-controlled facility with a 12-h light/dark cycle. A traumatic cataract model was established, as described previously 17. Briefly, male mice were anesthetized via intraperitoneal injection of 20 mL/kg body weight 1% sodium pentobarbital (Sigma, St. Louis, MO, USA; P3761), followed by topical application of compound tropicamide eye drops (Santen Pharmaceutical, Osaka, Japan) and oxybuprocaine hydrochloride (0.4%) eye drops (Santen Pharmaceutical, Osaka, Japan). After pupillary dilation, a small incision was made in the anterior capsule via a 33G needle inserted through the cornea in the right eye of the mouse. The injury depth was approximately 300 μm or one-fourth of the length of the blade part of the needle.^17^ Seven days after injury, a slit lamp examination was used to observe lens opacity. Moreover, eyes with opacity that occurred abruptly in the posterior cortical area were excluded because of the potential risk of severe uveitis.^49^ The laser-induced lens injury model mice were treated with the same anesthesia (20 mL/kg body weight of 1% sodium pentobarbital). After topical application of the compound tropicamide and oxybuprocaine hydrochloride eye drops, an Nd:YAG laser (5 mJ, 1 burst) was immediately applied to the lens anterior capsule, resulting in subcapsular gas bubble formation. Laser manipulation was performed by an experienced cataract specialist. For MLT treatment, the mice received a daily intraperitoneal administration of MLT at 10 mg/kg/day for 7 d.

### Lens epithelial explants collection, cell culture, and treatment

Human lens capsule samples with epithelial cell attachment were obtained from patients with senile cataracts at the Department of Ophthalmology at the First Affiliated Hospital of the Fourth Military Medical University. This study was approved by the Medical Ethics Committee of the First Affiliated Hospital of the Air Force Medical University (KY20212051-C-1). Patients with other ocular surgery histories or systemic disorders, such as diabetes and hypertensive disease, were excluded. During cataract surgery, an experienced cataract specialist performed the capsulorhexis maneuver to obtain lens capsule samples. The study was approved by the Ethics Committee of the Fourth Military Medical University in accordance with the Declaration of Helsinki, and written informed consent was obtained from each patient. Lens epithelial explants were washed in phosphate-buffered saline (PBS) supplemented with 1% penicillin and then cultured in a dish containing 20% fetal bovine serum (FBS) in Dulbecco's Modified Eagle's Medium (DMEM) at 37°C with 5% CO_2_. The cell culture medium was changed every other day. For ATF6 overexpression, lens epithelial explants from the same patient were split into two groups and treated separately with AAV-ATF6 or AAV-mCherry as a control. The human lens epithelial cell line HLE-B3 cells were purchased from the American Type Culture Collection (Manassas, VA, USA) and identified by Wuhan Pricella Biotechnology Co., Ltd. (Wuhan, China) via short tandem repeat (STR) profiling. Cells were cultured in DMEM supplemented with 10% FBS, 100 mg/mL streptomycin, and 100 IU/mL penicillin (Gibco, Grand Island, New York, USA). For induction of the UPR and treatment, HLE-B3 cells were incubated with tunicamycin (TM), a UPR inducer, at 4 μg/mL supplemented with or without 10 μM MLT (HY-B0075, MCE, Shanghai, China) or 6 μM Ceapin-A7 (HY-B1756, MCE, Shanghai, China).

### Immunofluorescence staining and H&E staining

For the animal experiments, the mice were sacrificed 7 d after injury. The peak time of the repair response was reached, and their eyes were enucleated for further analysis. Lens capsules were isolated, spread flat immediately under a microscope, and fixed in methanol for 2 h at 37℃. For human lens epithelial explants and cultured HLE-B3 plates, samples were fixed with 4% paraformaldehyde for 2 h at 4°C after being washed three times with PBS. The mouse and human samples were subsequently blocked and permeabilized with 1% Triton X-100 in 1% BSA for 2 h at room temperature and incubated with primary antibodies at 4°C overnight. Following three washes with PBS, the samples were incubated with fluorescently labeled secondary antibodies for 1 h at room temperature, followed by counterstaining with 4′,6-diamidino-2-phenylindole (DAPI). The primary antibodies used included antibodies against ATF6 (1:50, 66563-1-Ig, Proteintech, China), SNAI1 (1:100, 13099-1-AP, Proteintech, China), fibronectin (1:200, ab137720; Abcam, Cambridge, UK), and α-SMA (1:200, ab7817; Abcam). Capsule flat mounts and 3D image rebuilding were achieved via confocal laser scanning microscopy and Nikon Image NIS-Elements Viewer software. Serial tissue sections (5 µm) were deparaffinized, rehydrated, and then stained H&E. After staining, sections were dehydrated through increasing concentrations of ethanol and xylene. Finally mounted with a synthetic resinous mounting medium under a glass coverslip for microscopic evaluation.

### AAV vector construction and administration

The *ATF6* over-expression (OE) vector was constructed as previously described 50. The active ATF6 (39-373) fragment was selected to construct the *ATF6* OE vectors. A comparison of different adeno-associated virus (AAV) serotypes revealed that only the AAV2/DJ vector could successfully infect LECs (Figure S2A). To achieve long-term gain-of-function *in vivo*, ATF6 was cloned and inserted into AAV2/DJ vectors, which contained an expression cassette in the 3′ UTR of a red fluorescent protein (mCherry) reporter gene under the control of the cytomegalovirus (CMV) constitutive promoter (AAV2/DJ-h-ATF6-mCherry) (Figure S2B). Vectors with the same promoter and mCherry were used as controls (AAV2/DJ-CMV-mCherry; Hanbio Biotechnology, Shanghai, China). For *in vivo* transfection experiments, the anterior chamber of each eye of each mouse was injected with ∼3 × 10^9^ vg AAV2/DJ.

### Lentivirus production and infection

Oligonucleotides encoding short hairpin (sh) RNA were cloned and inserted into the pHBLV-U6-Puro vector to achieve lentiviral vector-driven ATF6 knockdown. Lentiviral vectors for *ATF6* OE were generated by inserting the transcript sequence of ATF6 into the pHBLV-CMV-mCherry-Puro vector (Hanbio Biotechnology, Shanghai, China). *SNAI1*-overexpressing lentiviral vectors (*SNAI1-*OE) were constructed from pLV-GFP-Puro vectors purchased from VigeneBio (Shandong, China). Oligonucleotides encoding short hairpin (sh) RNA (GGTGTGACTAACTATGCAA) were cloned and inserted into the pHBLV-U6-Puro vector to achieve lentiviral vector-driven SNAI1 knockdown (Hanbio Biotechnology, Shanghai, China). The fluorescence markers mCherry and GFP were utilized to assess the transfection efficiency of the lentiviral vectors *in vitro*. Following a one-week incubation period with the aforementioned vectors, the cells were subjected to selection with puromycin at a concentration of 2 μg/mL. (INVIVOGEN, Japan). Purified cells were used for downstream experiments.

### Dual-luciferase reporter assay

To investigate the influence of ATF6 on SNAI1 promoter activity, we cloned a series of potential promoters of SNAI1 (-2000/+100, -1500/-600, and -200/+100 regions relative to the known transcription start site) into the pGL3-luciferase vector (Sangon Biotech) to facilitate luciferase expression. Additionally, a gene fragment encoding the ATF6 protein was cloned and inserted into the pCDNA3.1 vector (Sangon Biotech). Human embryonic kidney (293T) cells were seeded in 24-well plates, cultured to subconfluence, and transfected with the above vectors via Lipo2000 (Invitrogen) according to the manufacturer's protocol. The Renilla luciferase reporter plasmid pRL-TK (Sangon Biotech) was also transfected as an internal control. To assess the influence of SNAI1 on the promoter activity of ATF6, similarly, we cloned and inserted a series of putative promoters of *ATF6* (the -2000/+130, -1300/+130, -800/+130, and -500/+130 regions, relative to the known transcription start site) into the pGL3-luciferase vector and cotransfected them into 293T cells with the pCDNA3.1 vector loaded with the encoding gene fragment of the SNAI1 protein. The luciferase activities were measured with a Dual-Glo Luciferase Assay Kit (#E1910, Promega, USA) 48 h after transfection. The results were normalized to Renilla luciferase activity, presented as ratios of Luc/Renilla activity, and acquired in triplicate from at least three different experiments.

### Wound-healing assay

A wound-healing assay was employed to assess lateral cell migration. Briefly, 3 × 10^5^ HLE-B3 cells per well were seeded in 6-well plates and cultured with complete medium to form cell monolayers at nearly 100% confluence. The cells were then scratched with a sterile pipette tip to create wounds of uniform size and incubated with 4 μg/mL TM supplemented with or without 10 μM MLT, and then cultured in a dish containing 1% FBS in DMEM at 37°C with 5% CO_2_. Images were captured at 0 and 24 h after scratching via microscopy. Wound closure was measured via ImageJ software (NIH, Bethesda, USA), and more than three random fields per sample were selected for assessment.

### Transwell assay

Transwell assays were used to evaluate vertical cell migration, and 24-well transwell plates with 8-µm pores were used in our experiments. HLE-B3 cells (4 × 10^4^ per well) were plated in the upper transwell chamber and treated with different drugs as mentioned above. After 24 h, noninvading cells were removed, and the cells in the lower chamber were fixed with 4% paraformaldehyde and stained with 0.25% crystal violet, followed by two washes with PBS. The cells that migrated to the lower surface of the upper chamber were examined, and relevant photos of four random visual fields per well were captured under a phase-contrast microscope. Stained cells in at least five random wells were counted in a blinded manner.

### Western blotting

For protein analysis, lens capsules and cellular proteins were harvested and homogenized in lysis buffer (RIPA, Biocolor, Shanghai, China) containing protease and phosphatase inhibitors (Beyotime, Shanghai, China). Nuclear proteins were isolated using a Nuclear and Cytoplasmic Protein Extraction Kit (Beyotime Biotechnology, Shanghai, China) according to the manufacturer's instructions. The protein concentration was determined with a bicinchoninic acid (BCA) assay (Beyotime). Equal concentrations of protein were loaded onto a sodium dodecyl sulfate‒polyacrylamide gel electrophoresis (SDS‒PAGE) gel for separation and then transferred to a polyvinylidene difluoride (PVDF) membrane. The membrane was blocked with 5% skim milk for 1 h at room temperature and incubated with different primary antibodies at 4°C overnight. The primary antibodies used included anti-p-PERK (29546-1-AP, Proteintech, China), anti-p-IRE1α (PA1-16927, Thermo Fisher Scientific, MA, USA), anti-CHOP (#5554, Cell Signaling Technology, MA, USA), anti-GRP78 (BIP, 11587-1-AP, Proteintech, China), anti-ATF6 (66563-1-Ig, Proteintech, China), anti-fibronectin (FN; ab137720; Abcam, Cambridge, UK), anti-E-cadherin (#14472, Cell Signaling Technology, MA, USA), anti-N-cadherin (22018-1-AP, Proteintech, China), anti-SNAI1 (13099-1-AP, Proteintech, China), anti-GAPDH (60004-1-Ig, Proteintech, China), anti-Histone H3 (#4499, Cell Signaling Technology, MA, USA) antibodies. After 3 washes, the membranes were incubated with horseradish peroxidase (HRP)-conjugated secondary antibodies at room temperature for 1 h. Finally, the membranes were visualized with a ChemiDoc Imaging System (Bio-Rad, USA).

### Statistical analysis

Statistical analyses were performed with GraphPad Prism software (version 8.0). Differences between the groups were analyzed with unpaired two-tailed Student's* t* test or one-way ANOVA with Tukey's post hoc tests when appropriate and as indicated in figure legends. *P* < 0.05 was considered significant.

## Supplementary Material

Supplementary figures and tables.

## Abbreviations

AAV: adeno-associated virus
ACTA2: actin alpha 2, smooth muscle
ASC: anterior subcapsular cataract
α-SMA: alpha-smooth muscle actin
ATF6: activating transcription factor 6
CHOP: C/EBP homologous protein
DDIT3: DNA damage inducible transcript 3
eIF2α: eukaryotic translation initiation factor 2α
EMT: epithelial‒mesenchymal transition
ER: endoplasmic reticulum
FN: fibronectin
HE: hematoxylin and eosin
IRE1α: inositol requiring enzyme 1α
HSPA5: heat shock protein family A member 5
LECs: lens epithelial cells
LP: lens puncture
LV: lentivirus
MLT: melatonin
PCO: posterior capsule opening
PERK: protein kinase R-like ER kinase
SNAI1: snail family transcriptional repressor 1
TF: transcription factor
TM: tunicamycin
UPR: unfolded protein response
