# Supplementary material for: Targeting the self-amplifying loop between ATF6 and SNAI1 to inhibit epithelial‒mesenchymal transition in fibrotic lesions
Source: Theranostics. 2025 Jul 11;15(16):7940–55. doi: 10.7150/thno.109442 (PMC12374540; doi:10.7150/thno.109442)
Supplement: Supplementary file 1 — Supplementary figures and tables. [file thnov15p7940s1.pdf]

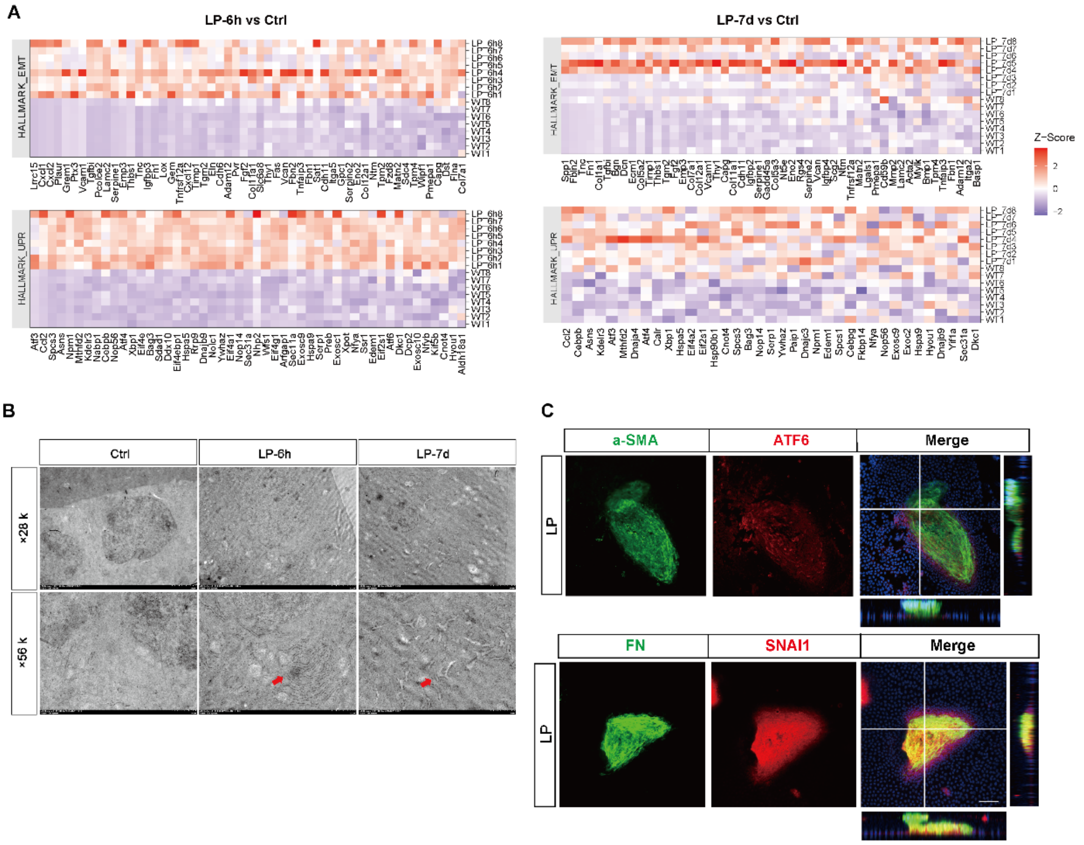

Figure S1

(A) Heatmaps of EMT and UPR related gene expressions based on the RNA-seq results of traumatic murine lens. (B) Sections of traumatic murine lens under transmission electron microscope. (C) 3D cross section images of whole-mount immunofluorescence staining with FN, α-SMA, SNAI1, and ATF6. Scale bars, 100μm.

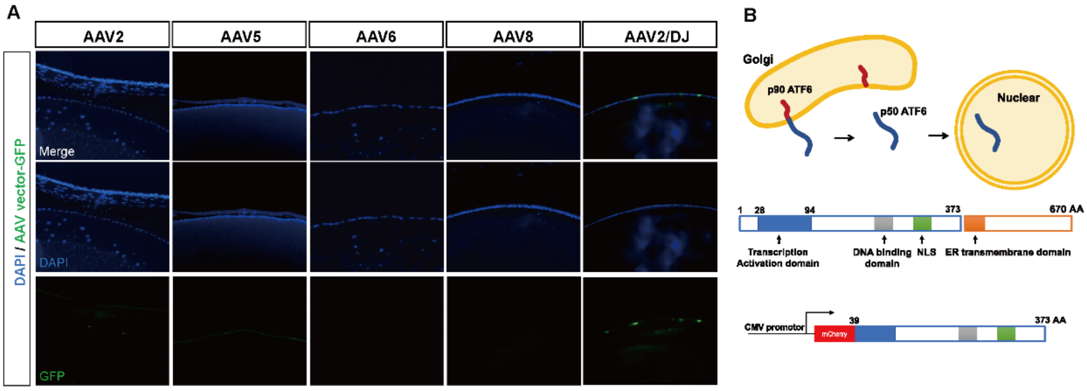

Figure S2

(A) Representative images of lens infected with different serotypes virus. Scale bars, 50μm. (B) AAV2/DJ-mCherry-ATF6 overexpression virus vector construct.

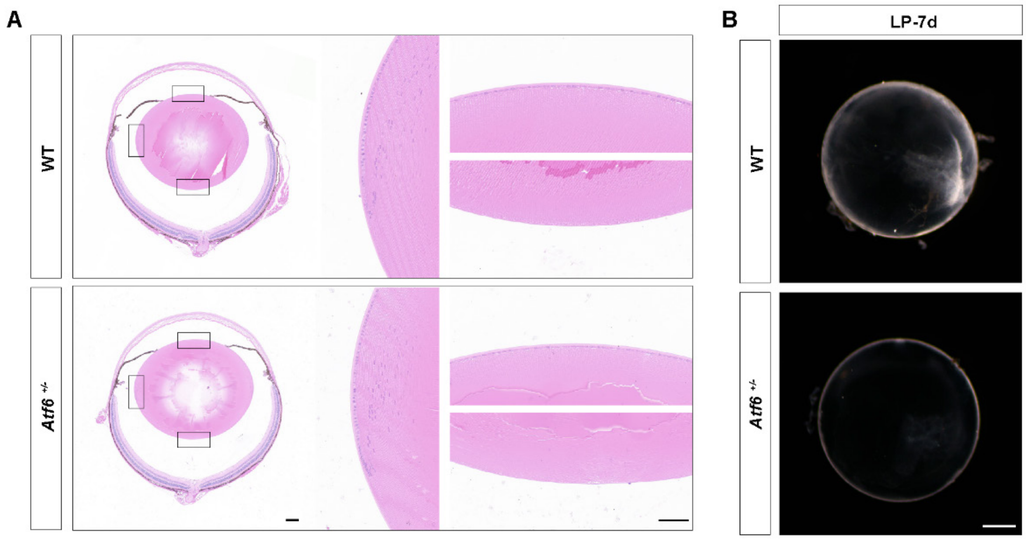

Figure S3

(A) Representative images of HE staining sections of lens in WT mice and *Atf6*$^{+/-}$ mice. Scale bars, 200μm& 50μm.(B) Representative images of lenses after 7 days of lens puncture in WT mice and *Atf6*$^{+/-}$ mice. Scale bars, 200μm.

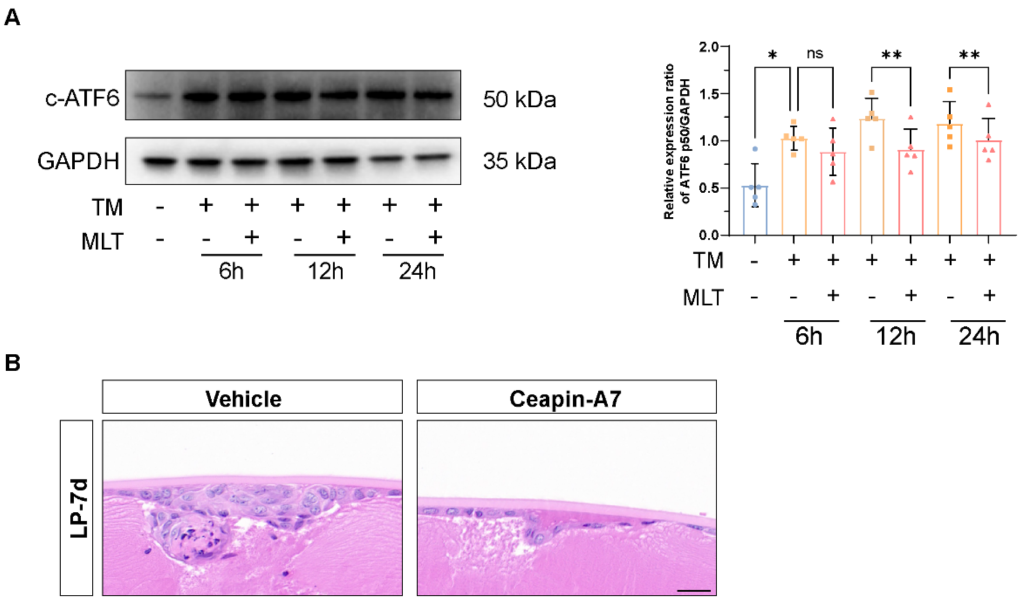

Figure S4

(A) Western blotting and quantification of c-ATF6 (n = 5). Data are presented as mean ± SEM; $^{*}p$ <0.05, $^{**}p$ <0.01, $^{***}p$ <0.001. (B) Representative images of HE-stained lens sections from LP mice and LP+Ceapin-A7-treated mice after 7 days of lens puncture. Scale bars, 50 µm.

Table S1

Predicted binding site of ATF6 on SNAI1 promoter region

Name	Sequence	Position (TSS-based)	Strand	Score
ATF6	GACGTCG	762	-	5.69
	TGACGCCC	528	-	0.41
	TGAGGTTG	1450	+	0.41
	TGACGCC	644	-	0.41
	GACGTCC	157	+	0.41

Table S2

Predicted binding site of SNAl1 on ATF6 promoter region

Name	Sequence	Position (TSS-based)	Strand	Score
SNAI1	GCACGTGTG	1039	+	8.298
	GCATGTGTG	1113	+	7.607
	GAAGGTGGG	567	+	7.335
	ACAGGTTTA	1424	-	6.811
	GCAAGTGAC	1686	-	6.505
	GAGGGTGTG	1027	-	6.190
	TCAGGTTTA	405	-	5.827
